# Optic Nerve Head Blood Flow Autoregulation during Changes in Arterial Blood Pressure in Healthy Young Subjects

**DOI:** 10.1371/journal.pone.0082351

**Published:** 2013-12-06

**Authors:** Agnes Boltz, Reinhard Told, Katarzyna J. Napora, Stefan Palkovits, René M. Werkmeister, Doreen Schmidl, Alina Popa-Cherecheanu, Gerhard Garhöfer, Leopold Schmetterer

**Affiliations:** 1 Department of Clinical Pharmacology, Medical University of Vienna, Vienna, Austria; 2 Center for Medical Physics and Biomedical Engineering, Medical University of Vienna, Vienna, Austria; 3 Department of Ophthalmology, Emergency University Hospital, Bucharest, Romania; Medical University Graz, Austria

## Abstract

**Aim:**

In the present study the response of optic nerve head blood flow to an increase in ocular perfusion pressure during isometric exercise was studied. Based on our previous studies we hypothesized that subjects with an abnormal blood flow response, defined as a decrease in blood flow of more than 10% during or after isometric exercise, could be identified.

**Methods:**

A total of 40 healthy subjects were included in this study. Three periods of isometric exercise were scheduled, each consisting of 2 minutes of handgripping. Optic nerve head blood flow was measured continuously before, during and after handgripping using laser Doppler flowmetry. Blood pressure was measured non-invasively in one-minute intervals. Intraocular pressure was measured at the beginning and the end of the measurements and ocular perfusion pressure was calculated as 2/3*mean arterial pressure –intraocular pressure.

**Results:**

Isometric exercise was associated with an increase in ocular perfusion pressure during all handgripping periods (p < 0.001). By contrast no change in optic nerve head blood flow was seen. However, in a subgroup of three subjects blood flow showed a consistent decrease of more than 10% during isometric exercise although their blood pressure values increased. In addition, three other subjects showed a consistent decline of blood flow of more than 10% during the recovery periods.

**Conclusion:**

Our data confirm previous results indicating that optic nerve head blood flow is autoregulated during an increase in perfusion pressure. In addition, we observed a subgroup of 6 subjects (15%) that showed an abnormal response, which is in keeping with our previous data. The mechanisms underlying this abnormal response remain to be shown.

## Introduction

Abnormalities in blood flow autoregulation at the posterior pole of the eye have been implicated in a variety of eye diseases including glaucoma, diabetes and age-related macular degeneration[1–6]. Although some support for this involvement has been provided, studies that investigated autoregulatory behavior in the human optic nerve head (ONH) are sparse. 

Autoregulation of ONH blood flow was reported in a variety of animal and human studies[7–16]. We recently reported on the behavior of ONH blood flow during both an increase and a decrease of ocular perfusion pressure (OPP) in healthy subjects[17]. In this study we did, however, also observe that there is a wide interindividual variability of this response and we were able to identify a subgroup of subjects with abnormal ONH blood flow autoregulation patterns. In this previous study we used a 6-minutes squatting period to increase blood pressure in order to achieve OPP values above the upper limit (approximately 64 mmHg in the sitting position) of autoregulation. 

This procedure of increasing OPP can, however, not be performed in the elderly or in patients with age-related eye disease. In the present study we set out to study the regulation of ONH blood flow during handgripping, a less demanding type of isometric exercise that has been used previously to study ocular blood flow autoregulation[18,19]. This was done in an effort to confirm and extend our previous data indicating that there are subjects with abnormal ONH blood flow autoregulation. In addition, we tried to get insight whether a protocol using repeated periods of handgripping may be an adequate approach to study ONH autoregulation in elderly patients with ocular disease.

## Materials and Methods

The present study was performed in adherence to the Declaration of Helsinki and the Good Clinical Practice (GCP) guidelines. The study protocol was approved by the Ethics Committee of the Medical University of Vienna and registered online prior to the beginning (Clinicaltrials.gov: NCT01663883, http://clinicaltrials.gov/ct2/show/NCT01663883). Forty healthy subjects aged between 19 and 35 years were included in this study. The nature of the study was explained to all subjects and written informed consent was obtained before participation. Each subject passed a screening examination that included medical history and physical examination. Subjects were excluded if they took any medication in the previous 3 weeks, were smokers, as well as if any abnormality was found as part of the pretreatment screening unless the investigators considered the abnormality to be clinically irrelevant. Moreover, an ophthalmic examination, including slit lamp biomicroscopy, indirect funduscopy and applanation tonometry, was performed. Inclusion criteria were normal ophthalmic findings, ametropia of less than 3 diopters, anisometropia of less than 1 diopter and intraocular pressure (IOP) < 20 mmHg.

The sample size calculation of the present study was based on the variability of our data as obtained in previous studies investigating ONH blood flow in our laboratory[17,20]. A repeated measures ANOVA model was underlying this sample size calculation. Given the variability in our previous experiments, an alpha error of 0.05 and a power of 0.80 a sample size of 40 healthy subjects was calculated to detect changes in ONH blood flow of 10%. Changes smaller than 10% were considered to be irrelevant.

### Experimental design

Subjects had to abstain from beverages containing xanthine derivates in the 12 hours before the trial day and arrived after a light meal and sleep for 7-8 hours. Dilatation of the pupil was obtained with topical tropicamide (Mydriaticum Agepha-Augentropfen, Vienna, Austria). After a resting period of at least 20 minutes in a sitting position, baseline measurements of systemic hemodynamics were performed. Stability of blood pressure and pulse rate was verified by repeated measurements before the actual experiments were started. Thereafter, ONH blood flow was measured continuously using laser Doppler flowmetry during rest and during isometric exercise with a handgrip. The following sequence was applied: rest-handgrip-rest-handgrip-rest-handgrip-rest while each period lasted for 2 minutes (Table 1). Systemic hemodynamics were measured every minute. IOP was assessed at the beginning and at the end of the study day.

**Table 1:** Experimental design.

BL 1	HG 1		RE 1	BL 2	HG 2		RE 2	BL 3	HG 3		RE 3
ONH blood flow measurement											
1-2	3	4	5	6	7	8	9	10	11	12	13-14
Time (minute)											

BL = baseline; HG = handgripping; RE = recovery

### Measurements

#### Systemic hemodynamics

Systolic blood pressure (SBP), diastolic blood pressure (DBP), and mean arterial blood pressure (MAP) were monitored on the upper arm by an automated oscillometric device. Pulse rate (PR) was automatically recorded from a finger pulse-oxymetric device (HP-CMS patient monitor, Hewlett Packard, Palo Alto, CA). The performance of this system has been reported previously[21].

#### Laser Doppler flowmetry

ONH blood flow was continuously measured employing a fundus camera-based laser Doppler flowmetry as described previously (Oculix 4000, Oculix, Arbaz, Switzerland)[17,22,23]. This technique uses coherent laser light to illuminate the vascularized tissue and uses the different scattering properties of static and moving cells to obtain blood flow data. Whereas light scattered by the moving red blood cells undergoes a frequency shift, light scattered by static tissue does not change light frequency, but leads to randomization of light directions impinging on red blood cells. Hence, red blood cells receive light from numerous random directions. The Doppler shift power spectrum is broadened by scattered light since the frequency shift depends not only on the velocity of the moving blood cells but also on the angle between the incident and the scattered light. Blood flow, velocity, and volume can be determined based on a theory of light scattering in tissue. Velocity is the mean velocity of the red blood cells moving in the sampled tissue proportional to the mean Doppler frequency shift. Volume is the number of moving red blood cells in the sampled tissue proportional to the amount of Doppler shifted light. Blood flow was calculated as the product of velocity and volume. Only data with a direct current (DC) value of ± 15% to the baseline value were included for analysis. The laser beam was directed towards the temporal neurovascular rim and any visible vessels within the scattering volume were avoided. The measurements were performed by two investigators, one who controlled the position of the laser beam to the subjects’ eye as well as the adequate fixation and the other controlled the signal on the computer.

#### Intraocular pressure and ocular perfusion pressure

Intraocular pressure (IOP) was measured using a slit-lamp mounted Goldmann applanation tonometer. In order to achieve local anesthesia of the cornea, one drop of 0.4 % benoxinate hydrochloride combined with 0.25 % sodium fluorescein was instilled before each measurement. Ocular perfusion pressure in the sitting position was calculated as 2/3*mean arterial blood pressure (MAP) – IOP[24].

#### Handgripping

During handgripping, the subject was instructed to rest his or her forearm on a table with the elbow at an angle of 90° during muscular contraction. The isometric handgrip was performed for 120 seconds at 75% of previously determined individual maximal voluntary contraction using a handgrip dynamometer.

### Data analysis

A repeated measures ANOVA model was used to analyze data. Post hoc analyses were done using planned comparisons. For this purpose the time effect was used to characterize the effect of handgripping on the outcome parameters. In addition, we analyzed individual blood flow traces to test the hypothesis that in some subjects blood flow may change during recovery as seen in the experiments presented in Schmidl et al. 2012[17]. As such we categorized data according to an increase or a decrease of more than 10% and analyzed during how many handgripping periods this was seen. In addition, we analyzed the reproducibility of the data. Two measures of reliability were calculated based on our measurements. The intraclass correlation coefficient (κ) was calculated based on an ANOVA model[25]. κ is calculated using the variance among subjects (ν_s_), the variance among measurements (ν_M_), and the residual error variance (ν_e_) and is given by

$$\kappa =\frac{\nu_{s}-\nu_{e}}{\nu_{s}+\nu_{e}+2\nu_{M}}$$

The higher the intra-class correlation coefficient the better is the reproducibility of the method. A κ of 1 reflects perfect reproducibility. In addition, the coefficients of variation were calculated. For this purpose, the standard deviation (SD) was calculated for each subject individually. By dividing the SD by the individual mean of ONH blood flow a coefficient of variation (CV) was calculated. As a measure of reproducibility the mean and SD of these individual CVs are presented. All calculations were done for the measurements at baseline, during handgripping and during the recovery period. For data description % changes over baseline were calculated. A p-value < 0.05 was considered the level of significance. Statistical analysis was carried out using CSS Statistica for Windows® (Statsoft Inc., Version 6.0, Tulsa, CA).

## Results

**Figure 1:**
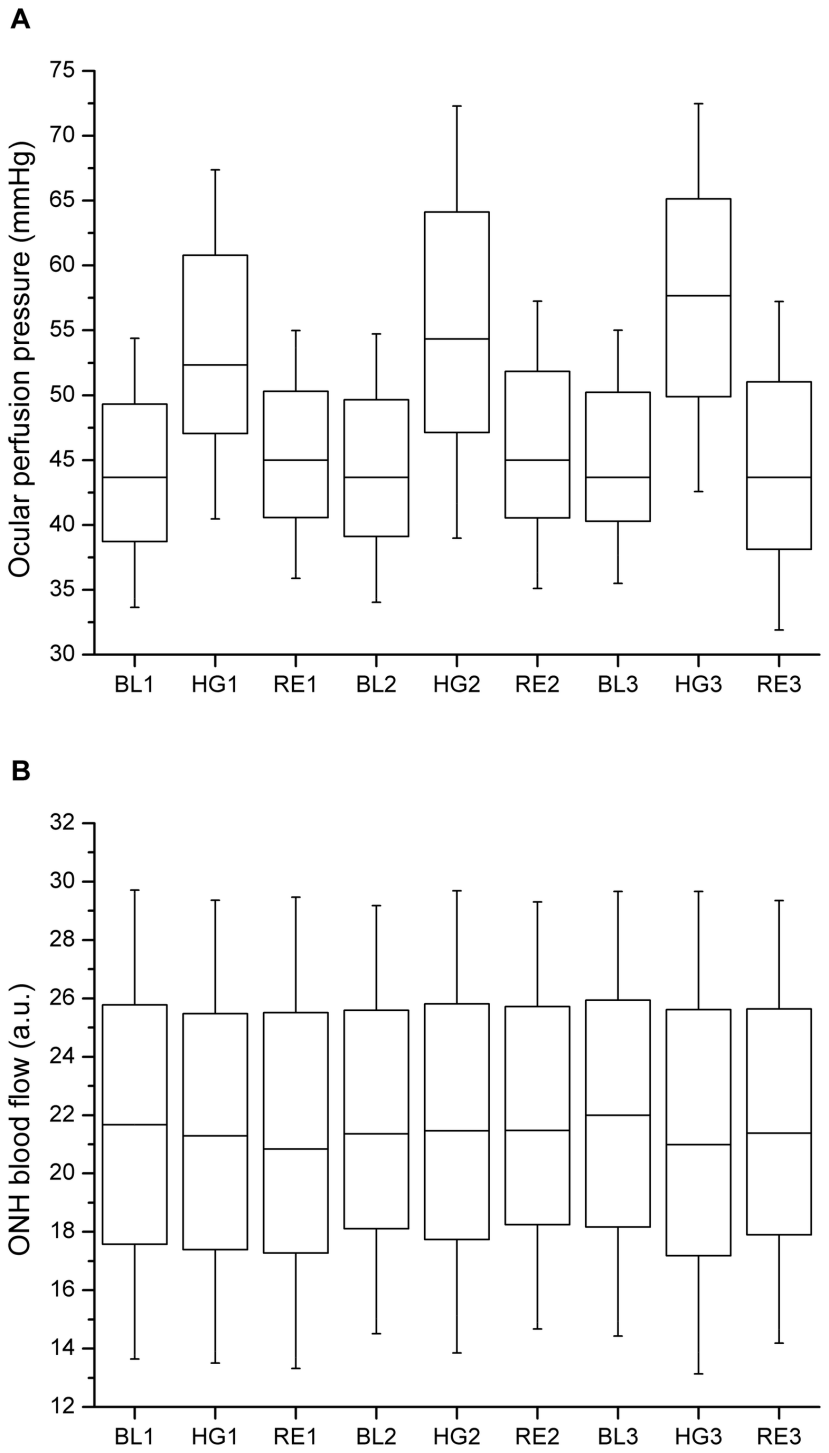
Effect of handgripping on ocular perfusion pressure (OPP, panel A) and optic nerve head blood flow (ONH blood flow, panel B). Three periods of isometric exercise were scheduled for each subject (n=39). Data are presented before during and after the 1^st^ handgripping experiment (BL1, HG1, RE1), before during and after the 2^nd^ handgripping experiment (BL2, HG2, RE2) and before during and after the 3^rd^ handgripping experiment (BL3, HG3, RE3). In the box-and-whisker blots the medians, standard deviations (SD) and 1.96 ± SDs are shown.

The baseline characteristics of the subjects are presented in Table 2. All participating subjects finished the study as scheduled and no adverse reactions were observed. In one subject, however, no adequate readings of ONH blood flow were obtained and data are therefore presented for 39 subjects only. The effect of isometric exercise on MAP and PR is shown in Table 3, the effect on OPP and ONH blood flow is depicted in Figure 1. Intraocular pressure did not change in the present experiments (baseline: 13.9 ± 1.4 mmHg; end of study: 13.5 ± 1.2 mmHg, p = 0.682). As expected, isometric exercise induced a significant increase in both MAP and PR during the three periods of isometric exercise (p < 0.001 each, ANOVA). As such a significant increase was also seen in OPP during isometric exercise (p < 0.001, ANOVA). By contrast no effect on ONH blood flow was observed (p = 0.112, ANOVA). 

**Table 2:** Demographic and baseline characteristics of the subjects participating in the study (n=40, mean ± SD).

Age (years)	23.9 ± 3.5
Sex (M/F)	20/20
Systolic blood pressure (mmHg)	117.8 ± 9.5
Diastolic blood pressure (mmHg)	66.0 ± 8.7
Mean arterial pressure (mmHg)	83.3 ± 7.3
Pulse rate (beats per minute)	71.1 ± 13.4
Intraocular pressure (mmHg)	13.9 ± 1.4
Ocular perfusion pressure (mmHg)	44.0 ± 5.3
Optic nerve head blood flow (arbitrary units)	21.2 ± 6.3

**Table 3:** Effects of isometric exercise (handgripping, 3 periods) on mean arterial pressure, pulse rate, ocular perfusion pressure, and optic nerve head blood flow in all patients (n=39, means ± SD).

	Before 1^st^ period	During 1^st^ period	After 1^st^ period	Before 2^nd^ period	During 2^nd^ period	After 2^nd^ period	Before 3^rd^ period	During 3^rd^ period	After 3^rd^ period
MAP (mmHg)	83.7 ± 7.5	96.7 ± 10.2	84.2 ± 7.7	85.7 ± 7.4	98.7 ± 11.4	84.5 ± 7.7	83.9 ± 7.1	99.2 ± 12.0	82.5 ± 7.0
PR (beats min-1)	71.0 ± 13.2	81.8 ± 13.6	72.0 ± 12.2	72.6 ± 11.8	82.6 ± 15.7	72.7 ± 13.2	72.7 ± 12.5	84.0 ± 15.7	72.7 ± 13.0
OPP (mmHg)	44.0 ± 5.2	53.5 ± 6.8	45.3 ± 4.8	44.3 ± 5.2	55.3 ± 8.4	46.0 ± 5.6	45.2 ± 4.9	57.1 ± 7.5	44.6 ± 6.4
ONHBF (a.u.)	21.7 ± 4.1	21.4 ± 4.0	21.4 ± 4.1	21.9 ± 3.7	21.8 ± 4.0	22.0 ± 3.7	22.1 ± 3.9	21.4 ± 4.2	21.8 ± 3.9

MAP = mean arterial pressure; PR = pulse rate, OPP = ocular perfusion pressure; ONHBF = optic nerve head blood flow; a.u. = arbitrary units.

Reproducibility was separately studied for baseline ONH blood flow data, for ONH blood flow data during isometric exercise and for ONH blood flow data during the recovery period. The intraclass correlation coefficient was 0.91, 0.86 and 0.88 for baseline, isometric exercise and recovery period, respectively. The good reproducibility of our data is also mirrored in the coefficients of variation, which were 7.0% at baseline, 10.1% during isometric exercise and 8.8% in the recovery period. 

The frequency of blood flow changes of more than 10% is summarized in Table 4. Three subjects showed a decrease of more than 10% in ONH blood flow during recovery at all 3 periods of isometric exercise. Unexpectedly, however, we also saw three other subjects who consistently showed an ONH blood flow reduction of more than 10% during isometric exercise. In some subjects an increase in ONH blood flow of more than 10% was seen either during or after isometric exercise during single periods. However, in none of the participating subjects ONH blood flow consistently increased by more than 10% during or after handgripping. The individual data of the subjects that showed a consistent abnormal response either during exercise or during recovery are presented in Table 5. 

**Table 4:** Frequency of abnormal blood flow changes of more than 10% (n=39).

**Change in optic nerve head blood flow of > 10%**	**Number of subjects**
**During handgripping**	
Decrease of more than 10% during 1, 2, or all 3 periods	3/0/3
Increase of more than 10% during 1, 2, or all 3 periods	6/0/0
**During recovery**	
Decrease of more than 10% during 1, 2, or all 3 periods	0/0/3
Increase of more than 10% during 1, 2, or all 3 periods	6/0/0

**Table 5:** Effects of isometric exercise (handgripping, 3 periods) on mean arterial pressure, pulse rate, ocular perfusion pressure, and optic nerve head blood flow in the subgroup of subjects that showed abnormal blood flow patterns either during (n=3) of after (n=3) isometric exercise defined as a decrease of more than 10% (individual data are presented as % change from baseline).

	Exercise 1	Recovery 1	Exercise 2	Recovery 2	Exercise 3	Recovery 3
Subjects with a consistent decrease in ONH blood flow during all the periods of exercise						
Subject 1						
MAP (mmHg)	14.2	2.3	15.6	2.1	21.1	-4.8
PR (beats min-1)	13.0	-1.9	17.6	4.9	20.8	-1.3
OPP (mmHg)	21.1	3.0	24.0	2.8	28.9	-6.9
ONHBF (a.u.)	-11.2	1.0	-14.3	2.7	-35.5	-7.3
Subject 2						
MAP (mmHg)	16.0	-4.3	12.9	-5.9	18.0	4.9
PR (beats min-1)	12.3	2.9	9.8	3.9	14.1	4.6
OPP (mmHg)	22.9	-5.9	17.4	-7.2	25.4	6.9
ONHBF (a.u.)	-32.3	1.5	-18.0	1.3	-31.7	2.4
Subject 3						
MAP (mmHg)	8.0	2.8	9.5	1.0	11.7	-0.7
PR (beats min-1)	12.9	8.1	14.2	-5.9	16.4	0.0
OPP (mmHg)	12.0	4.2	13.9	1.6	16.5	-1.1
ONHBF (a.u.)	-15.6	2.9	-11.4	14.1	-20.4	-5.3
Subjects with a consistent decrease in ONH blood flow during all the recovery periods						
Subject 4						
MAP (mmHg)	17.2	-3.9	19.1	-2.9	21.4	-6.0
PR (beats min-1)	21.8	0.8	22.5	9.2	17.9	-5.1
OPP (mmHg)	23.9	-5.9	26.0	-4.5	28.9	-8.9
ONHBF (a.u.)	2.4	-26.6	-5.0	-19.7	7.3	-18.2
Subject 5						
MAP (mmHg)	9.3	4.3	10.7	-1.8	10.5	-7.1
PR (beats min-1)	8.9	-4.5	9.3	-6.1	12.7	-7.9
OPP (mmHg)	14.2	6.8	15.9	-3.0	15.6	-10.0
ONHBF (a.u.)	-2.1	-22.9	-2.7	-19.7	5.5	-17.8
Subject 6						
MAP (mmHg)	14.8	-3.8	12.0	7.0	17.9	-0.8
PR (beats min-1)	21.9	9.1	22.9	3.0	19.2	0.0
OPP (mmHg)	21.9	5.7	17.8	10.3	26.3	-1.3
ONHBF (a.u)	-11.1	-21.7	5.1	-11.2	7.0	-10.6

MAP = mean arterial pressure, PR = pulse rate, OPP = ocular perfusion pressure; ONHBF = optic nerve head blood flow; a.u. = arbitrary units.

## Discussion

The present study confirms previous data showing that in humans ONH blood flow is autoregulated. During a handgrip-induced increase in OPP of approximately 15 mmHg ONH blood flow did not change indicating that the ONH vasculature vasoconstricts in response to the increase in perfusion pressure thereby keeping perfusion constant. In keeping with our previous data[17] some individuals showed abnormal ONH blood flow autoregulation patterns. In our previous study we observed in some subjects that during squatting ONH blood flow fell below baseline levels during OPP fluctuations, although absolute OPP was considerably higher than at baseline. The present study was designed to confirm and extend these data.

As such we chose three repeated periods of a less demanding form of isometric exercise with intermitting resting periods. We hypothesized that in some subjects ONH blood flow might fall below baseline. To quantify whether this is consistently seen we defined a more than 10% decrease as an abnormal response. Indeed, we observed a subgroup of 3 healthy subjects in whom ONH blood flow values fell below baseline during the recovery periods although blood pressure was not lower than baseline. In addition, three other subjects showed a consistent decline of ONH blood flow during isometric exercise although their blood pressure values increased.

Whereas these data are well compatible with our previous studies[17], the reason for this behavior remains unclear. In autoregulation the vasculature produces vasoconstrictors to counteract the increase in perfusion pressure in order to keep blood flow constant[26–28]. As such it is one possibility that subjects with abnormal autoregulation flow patterns show an overproduction of vasoconstrictors. This hypothesis is, however, difficult to prove, because the mechanisms underlying ONH autoregulation are largely unknown. We have recently shown that endothelin_A_ receptor blockade modifies the response of ONH blood flow to isometric exercise[29] as it does in the choroid[30]. Nitric oxide (NO) synthase inhibition which alters the response of choroidal blood flow to isometric exercise[31] does not alter the response in the ONH[32] although animal data suggest an involvement of NO in ONH autoregulation[10,33]. In addition, a recent study indicates that astrocytes are involved in the autoregulatory response of the ONH in face of changes in OPP[11]. A contribution of the central nervous system to ONH autoregulation can be excluded, because the anterior ONH vasculature lacks autonomic innervation[34].

Whether abnormal ONH blood flow response patterns to changes in OPP predispose to ocular vascular diseases is unknown. For several common eye diseases such as diabetic retinopathy[2,35] and glaucoma[5,6,36] abnormalities in ocular blood flow autoregulation have been reported. In primary open glaucoma reduced OPP is a risk factor for the disease[6,37–39] and it has been hypothesized that ONH blood flow falls below the lower limit of autoregulation during periods of low blood pressure or high IOP[26]. Whether abnormal ONH blood flow response patterns, as observed in the present study, are more frequent in glaucoma is unknown. In the choroid glaucoma patients show abnormal autoregulation in response to handgripping as compared to healthy subjects[19]. The protocol used in the present study may be sufficient to study ONH autoregulation also in patients with vascular disease.

Several limitations need to be considered for this study. The selection of a 10% change in ONH blood flow to define an abnormal response is arbitrary. Unfortunately there is currently no data available indicating which degree of ONH ischemia is detrimental for the ONH tissue. Our reproducibility data indicate, however, that 10% changes in ONH blood flow can be detected with the present protocol. Interestingly, reproducibility data were better than those previously reported from our laboratory several years ago[40,41]. Several factors may contribute to this observation. On the one hand our previous study assessed reproducibility over several hours, whereas in the present study subjects did not remove their heads from the headrest during the entire period, making positioning of the patient in front of the instrument less of a problem. In addition, we did some minor modifications to the original instrument including an improved fixation target and an alternative coupling of the light source to the fundus camera. We do not know whether such excellent reproducibility can also be achieved in elderly patient groups. It is also unclear whether the margin of 10% selected in this study is clinically meaningful. For this purpose studies need to be performed in relevant patient groups investigating specificity and sensitivity.

Another issue relates to the definition of the recovery period and the schedule of our protocol. We decided to keep the experiment as short as possible and therefore defined the first minute after exercise as recovery and the second minute as baseline for the following period of exercise. In those patients who had a decrease in ONH blood flow during recovery (Subjects 4-6 in Table 5) we observed that blood flow values were typically back at baseline after 60 seconds and that the decrease in ONH blood flow peaked approximately 20-30 seconds after cessation of exercise. We did not measure IOP during the handgripping experiments, but only at the beginning and the end of the study day. We deem it, however, unlikely that this represents a major problem, because even in our previous studies where blood pressure was raised to much higher levels using squatting the effects on IOP were considerably small[29–32,42]. In addition, there is evidence that LDF only assesses the anterior parts of the ONH vasculature[22]. As such our results cannot necessarily be applied to the posterior ONH, a limitation that has been discussed in more detail in a previous paper[17].

In conclusion, our data indicate that ONH blood flow is well autoregulated when OPP is increased by isometric exercise by approximately 15 mmHg. In three subjects, however, we observed that ONH blood flow decreased by more than 10% during all periods of isometric exercise although OPP was elevated. In three other subjects ONH blood flow decreased by more than 10% in the recovery period that followed isometric exercise. Whereas this is in keeping with our previous data both, the reason for this behavior and the potential consequences for the incidence of ocular diseases are unclear. 

## References

[1] Toda N, Nakanishi-Toda M (2007) Nitric oxide: ocular blood flow, glaucoma, and diabetic retinopathy. Prog Retin Eye Res 26: 205-238. doi:10.1016/j.preteyeres.2007.01.004. PubMed: 17337232. 17337232

[2] Pemp B, Schmetterer L (2008) Ocular blood flow in diabetes and age-related macular degeneration. Can J Ophthalmol 43: 295-301. doi:10.3129/i08-049. PubMed: 18443612. 18443612

[3] Feigl B (2009) Age-related maculopathy - linking aetiology and pathophysiological changes to the ischaemia hypothesis. Prog Retin Eye Res 28: 63-86. doi:10.1016/j.preteyeres.2008.11.004. PubMed: 19070679. 19070679

[4] Kur J, Newman EA, Chan-Ling T (2012) Cellular and physiological mechanisms underlying blood flow regulation in the retina and choroid in health and disease. Prog Retin Eye Res 31: 377-406. doi:10.1016/j.preteyeres.2012.04.004. PubMed: 22580107. 22580107 PMC3418965

[5] Schmidl D, Garhofer G, Schmetterer L (2011) The complex interaction between ocular perfusion pressure and ocular blood flow - relevance for glaucoma. Exp Eye Res 93: 141-155. doi:10.1016/j.exer.2010.09.002. PubMed: 20868686. 20868686

[6] Cherecheanu AP, Garhofer G, Schmidl D, Werkmeister R, Schmetterer L (2013) Ocular perfusion pressure and ocular blood flow in glaucoma. Curr Opin Pharmacol 13: 36-42. doi:10.1016/j.coph.2012.09.003. PubMed: 23009741. 23009741 PMC3553552

[7] Pillunat LE, Anderson DR, Knighton RW, Joos KM, Feuer WJ (1997) Autoregulation of human optic nerve head circulation in response to increased intraocular pressure. Exp Eye Res 64: 737-744. doi:10.1006/exer.1996.0263. PubMed: 9245904. 9245904

[8] Riva CE, Hero M, Titze P, Petrig B (1997) Autoregulation of human optic nerve head blood flow in response to acute changes in ocular perfusion pressure. Graefes Arch Clin Exp Ophthalmol 235: 618-626. doi:10.1007/BF00946937. PubMed: 9349945. 9349945

[9] Movaffaghy A, Chamot SR, Petrig BL, Riva CE (1998) Blood flow in the human optic nerve head during isometric exercise. Exp Eye Res 67: 561-568. doi:10.1006/exer.1998.0556. PubMed: 9878218. 9878218

[10] Okuno T, Oku H, Sugiyama T, Yang Y, Ikeda T (2002) Evidence that nitric oxide is involved in autoregulation in optic nerve head of rabbits. Invest Ophthalmol Vis Sci 43: 784-789. PubMed: 11867599. 11867599

[11] Shibata M, Sugiyama T, Kurimoto T, Oku H, Okuno T et al. (2012) Involvement of glial cells in the autoregulation of optic nerve head blood flow in rabbits. Invest Ophthalmol Vis Sci 53: 3726-3732. doi:10.1167/iovs.11-9316. PubMed: 22589427. 22589427

[12] Weigert G, Findl O, Luksch A, Rainer G, Kiss B et al. (2005) Effects of moderate changes in intraocular pressure on ocular hemodynamics in patients with primary open-angle glaucoma and healthy controls. Ophthalmology 112: 1337-1342. doi:10.1016/j.ophtha.2005.03.016. PubMed: 16024084. 16024084

[13] Iester M, Torre PG, Bricola G, Bagnis A, Calabria G (2007) Retinal blood flow autoregulation after dynamic exercise in healthy young subjects. Ophthalmologica 221: 180-185. doi:10.1159/000099298. PubMed: 17440280. 17440280

[14] Liang Y, Fortune B, Cull G, Cioffi GA, Wang L (2010) Quantification of dynamic blood flow autoregulation in optic nerve head of rhesus monkeys. Exp Eye Res 90: 203-209. doi:10.1016/j.exer.2009.10.009. PubMed: 19853603. 19853603

[15] Piper C, Fortune B, Cull G, Cioffi GA, Wang L (2013) Basal blood flow and autoregulation changes in the optic nerve of rhesus monkeys with idiopathic bilateral optic atrophy. Invest Ophthalmol Vis Sci 54: 714-721. doi:10.1167/iovs.12-9773. PubMed: 23287792. 23287792 PMC3559073

[16] Shiga Y, Shimura M, Asano T, Tsuda S, Yokoyama Y et al. (2013) The influence of posture change on ocular blood flow in normal subjects, measured by laser speckle flowgraphy. Curr Eye Res 38: 691-698. doi:10.3109/02713683.2012.758292. PubMed: 23654357. 23654357

[17] Schmidl D, Boltz A, Kaya S, Werkmeister R, Dragostinoff N et al. (2012) Comparison of choroidal and optic nerve head blood flow regulation during changes in ocular perfusion pressure. Invest Ophthalmol Vis Sci 53: 4337-4346. doi:10.1167/iovs.11-9055. PubMed: 22661477. 22661477

[18] Schmetterer L, Dallinger S, Findl O, Strenn K, Graselli U et al. (1998) Noninvasive investigations of the normal ocular circulation in humans. Invest Ophthalmol Vis Sci 39: 1210-1220. PubMed: 9620081. 9620081

[19] Portmann N, Gugleta K, Kochkorov A, Polunina A, Flammer J et al. (2011) Choroidal blood flow response to isometric exercise in glaucoma patients and patients with ocular hypertension. Invest Ophthalmol Vis Sci 52: 7068-7073. doi:10.1167/iovs.11-7758. PubMed: 21828157. 21828157

[20] Polak K, Luksch A, Berisha F, Fuchsjaeger-Mayrl G, Dallinger S et al. (2007) Altered nitric oxide system in patients with open-angle glaucoma. Arch Ophthalmol 125: 494-498. doi:10.1001/archopht.125.4.494. PubMed: 17420369. 17420369

[21] Wolzt M, Schmetterer L, Rheinberger A, Salomon A, Unfried C et al. (1995) Comparison of non-invasive methods for the assessment of haemodynamic drug effects in healthy male and female volunteers: sex differences in cardiovascular responsiveness. Br J Clin Pharmacol 39: 347-359. doi:10.1111/j.1365-2125.1995.tb04462.x. PubMed: 7640140. 7640140 PMC1365121

[22] Riva CE, Geiser M, Petrig BL (2010) Ocular blood flow assessment using continuous laser Doppler flowmetry. Acta Ophthalmol 88: 622-629. doi:10.1111/j.1755-3768.2009.01621.x. PubMed: 19860779. 19860779

[23] Riva CE, Harino S, Petrig BL, Shonat RD (1992) Laser Doppler flowmetry in the optic nerve. Exp Eye Res 55: 499-506. doi:10.1016/0014-4835(92)90123-A. PubMed: 1426079. 1426079

[24] Robinson F, Riva CE, Grunwald JE, Petrig BL, Sinclair SH (1986) Retinal blood flow autoregulation in response to an acute increase in blood pressure. Invest Ophthalmol Vis Sci 27: 722-726. PubMed: 3700021. 3700021

[25] Bartko JJ, Carpenter WT Jr (1976) On the methods and theory of reliability. J Nerv Ment Dis 163: 307-317. doi:10.1097/00005053-197611000-00003. PubMed: 978187. 978187

[26] Flammer J, Mozaffarieh M (2008) Autoregulation, a balancing act between supply and demand. Can J Ophthalmol 43: 317-321. doi:10.1139/I08-056. PubMed: 18493273. 18493273

[27] Claassen JA, Zhang R (2011) Cerebral autoregulation in Alzheimer's disease. J Cereb Blood Flow Metab 31: 1572-1577. doi:10.1038/jcbfm.2011.69. PubMed: 21540872. 21540872 PMC3137479

[28] Tzeng YC, Ainslie PN (2013) Blood pressure regulation IX: cerebral autoregulation under blood pressure challenges. Eur J Appl Physiol. 10.1007/s00421-013-2667-y PMC3929776 23737006

[29] Boltz A, Schmidl D, Werkmeister RM, Lasta M, Kaya S et al. (2013) Role of endothelin-A receptors in optic nerve head red cell flux regulation during isometric exercise in healthy humans. Am J Physiol Heart Circ Physiol 304: H170-H174. doi:10.1152/ajpheart.00408.2012. PubMed: 23103498. 23103498

[30] Fuchsjager-Mayrl G, Luksch A, Malec M, Polska E, Wolzt M et al. (2003) Role of endothelin-1 in choroidal blood flow regulation during isometric exercise in healthy humans. Invest Ophthalmol Vis Sci 44: 728-733. 12556405 10.1167/iovs.02-0372

[31] Luksch A, Polska E, Imhof A, Schering J, Fuchsjager-Mayrl G et al. (2003) Role of NO in choroidal blood flow regulation during isometric exercise in healthy humans. Invest Ophthalmol Vis Sci 44: 734-739. doi:10.1167/iovs.02-0177. PubMed: 12556406. 12556406

[32] Schmidl D, Boltz A, Kaya S, Lasta M, Pemp B et al. (2013) Role of nitric oxide in optic nerve head blood flow regulation during isometric exercise in healthy humans. Invest Ophthalmol Vis Sci 54: 1964-1970. doi:10.1167/iovs.12-11406. PubMed: 23439596. 23439596

[33] Takayama H, Hamner CE, Caccitolo JA, Hisamochi K, Pearson PJ et al. (2003) A novel antioxidant, EPC-K1, stimulates endothelial nitric oxide production and scavenges hydroxyl radicals. Circ J 67: 1046-1052. doi:10.1253/circj.67.1046. PubMed: 14639022. 14639022

[34] Mackenzie PJ, Cioffi GA (2008) Vascular anatomy of the optic nerve head. Can J Ophthalmol 43: 308-312. doi:10.3129/i08-042. PubMed: 18443611. 18443611

[35] Ciulla TA, Harris A, Latkany P, Piper HC, Arend O et al. (2002) Ocular perfusion abnormalities in diabetes. Acta Ophthalmol Scand 80: 468-477. doi:10.1034/j.1600-0420.2002.800503.x. PubMed: 12390156. 12390156

[36] Moore D, Harris A, Wudunn D, Kheradiya N, Siesky B (2008) Dysfunctional regulation of ocular blood flow: A risk factor for glaucoma? Clin Ophthalmol 2: 849-861. 19668439 10.2147/opth.s2774 PMC2699797

[37] Leske MC (2009) Ocular perfusion pressure and glaucoma: clinical trial and epidemiologic findings. Curr Opin Ophthalmol 20: 73-78. doi:10.1097/ICU.0b013e32831eef82. PubMed: 19240538. 19240538 PMC2662722

[38] Costa VP, Arcieri ES, Harris A (2009) Blood pressure and glaucoma. Br J Ophthalmol 93: 1276-1282. doi:10.1136/bjo.2008.149047. PubMed: 19336425. 19336425

[39] He Z, Vingrys AJ, Armitage JA, Bui BV (2011) The role of blood pressure in glaucoma. Clin Exp Optom 94: 133-149. doi:10.1111/j.1444-0938.2010.00564.x. PubMed: 21255075. 21255075

[40] Luksch A, Lasta M, Polak K, Fuchsjager-Mayrl G, Polska E et al. (2009) Twelve-hour reproducibility of retinal and optic nerve blood flow parameters in healthy individuals. Acta Ophthalmol 87: 875-880. 18976308 10.1111/j.1755-3768.2008.01388.x

[41] Polska E, Polak K, Luksch A, Fuchsjager-Mayrl G, Petternel V et al. (2004) Twelve hour reproducibility of choroidal blood flow parameters in healthy subjects. Br J Ophthalmol 88: 533-537. doi:10.1136/bjo.2003.028480. PubMed: 15031172. 15031172 PMC1772102

[42] Polska E, Simader C, Weigert G, Doelemeyer A, Kolodjaschna J et al. (2007) Regulation of choroidal blood flow during combined changes in intraocular pressure and arterial blood pressure. Invest Ophthalmol Vis Sci 48: 3768-3774. doi:10.1167/iovs.07-0307. PubMed: 17652750. 17652750

